# Truncated TALE-FP as DNA Staining Dye in a High-salt Buffer

**DOI:** 10.1038/s41598-019-53722-0

**Published:** 2019-11-20

**Authors:** Eunji Shin, Woojung Kim, Seonghyun Lee, Jaeyoung Bae, Sanggil Kim, Wooseok Ko, Ho Seong Seo, Sangyong Lim, Hyun Soo Lee, Kyubong Jo

**Affiliations:** 1 Department of Chemistry and Interdisciplinary Program of Integrated Biotech, Sogang University, 1 Shinsudong, Mapogu, Seoul, 04107 Korea; 2 Research Division for Biotechnology, Korea Atomic Energy Research Institute, Jeongeup, 580-185 Korea

**Keywords:** Single-molecule biophysics, Bioanalytical chemistry

## Abstract

Large DNA molecules are a promising platform for *in vitro* single-molecule biochemical analysis to investigate DNA-protein interactions by fluorescence microscopy. For many studies, intercalating fluorescent dyes have been primary DNA staining reagents, but they often cause photo-induced DNA breakage as well as structural deformation. As a solution, we previously developed several fluorescent-protein DNA-binding peptides or proteins (FP-DBP) for reversibly staining DNA molecules without structural deformation or photo-induced damage. However, they cannot stain DNA in a condition similar to a physiological salt concentration that most biochemical reactions require. Given these concerns, here we developed a salt-tolerant FP-DBP: truncated transcription activator-like effector (tTALE-FP), which can stain DNA up to 100 mM NaCl. Moreover, we found an interesting phenomenon that the tTALE-FP stained DNA evenly in 1 × TE buffer but showed AT-rich specific patterns from 40 mM to 100 mM NaCl. Using an assay based on fluorescence resonance energy transfer, we demonstrated that this binding pattern is caused by a higher DNA binding affinity of tTALE-FP for AT-rich compared to GC-rich regions. Finally, we used tTALE-FP in a single molecule fluorescence assay to monitor real-time restriction enzyme digestion of single DNA molecules. Altogether, our results demonstrate that this protein can provide a useful alternative as a DNA stain over intercalators.

## Introduction

Large DNA molecules have been a versatile platform to investigate a variety of genomic, epigenetic, biochemical, and biophysical studies^1^. For genome analysis, Optical Mapping and nanocoding have been representative single-molecule analytical systems to obtain DNA physical maps^2–6^. They have been applied for many genome projects to aid genome assembly with sequencing^7,8^, as well as to reveal large scale structural variations of the genome^9,10^. Single-molecule DNA maps have also been utilized for the rapid identification of DNA molecules without sequencing^11,12^. Alternatively, elongated DNA molecules provide a platform to visualize biochemical information, such as chemical modifications^13,14^ and DNA damage at the single-molecule level^15–18^. There have been many studies conducted to monitor enzymatic motions and functions on a large elongated DNA molecule, such as the dynamics of DNA replication forks^19,20^, the movement of DNA translocases^21^, and the role of polymerase subunits^22^.

Most of those studies have commonly used intercalating fluorescent dyes such as YOYO-1^23^, SYTOX, and SYBR^24^. However, these organic dyes present major drawbacks for staining DNA molecules^25^. For example, their intercalation into DNA induces photodamage under continuous laser illumination through the formation of radical intermediates that create single-, or double-stranded breaks in DNA molecules^26,27^. This intercalation also alters the structure and mechanical properties of DNA^28^, which can perturb enzymatic reactions on a DNA molecule, in bulk reactions^29–31^, as well as at the single-molecule level^32,33^.

As a solution, we have previously reported the development of FP-tagged DNA-binding peptides (FP-DBPs) as new staining reagents^25,34–36^. FP-DBPs do not cause photo-induced DNA cleavage or structural deformation. Moreover, it is possible to control DNA staining reversibly by the pH shift or adjusting salt concentration. This feature allows DNA molecules to be monitored as follows: DNA staining, destaining, biochemical reaction, and another staining to watch the result of the reaction. However, some scientists personally asked whether it is possible to stain DNA with FP-DBP in their reaction buffers that contain a salt concentration similar to a physiological condition, such as phosphate-buffered saline (PBS). Unfortunately, we found that FP-DBPs reported so far were not compatible with these reaction buffers.

To overcome the issue of salt-tolerance, we attempted to develop a novel FP-DBP using a gene of transcription activator-like effector (TALE). TALE-FP has been used for various applications to visualize their interactions with DNA within the cell nucleus^37,38^. Besides, TALE linked to a nuclease domain (TALEN) is one of the well-known genome-editing tools^39^. From these previous studies, we noticed that TALE-FP and TALEN are capable of binding DNA within a cell nucleus that generally contains relatively high-salt concentrations. For example, a previous paper reported the salt concentration of 150 mM NaCl and 260 mM KCl in a cell nucleus^40^. Accordingly, TALE-FP can be a promising candidate for staining DNA in a high-salt solution.

We added an FP gene at the C-terminus of the 7-mer DNA binding domain from a 20-mer target sequence TALEN gene^41^. As expected, this short-length truncated TALE-FP (tTALE-FP) showed salt-tolerant DNA staining capability. More specifically, this protein stained DNA evenly in Tris-EDTA buffer (1 × TE). The increase of NaCl concentration preserved tTALE-FPs at AT-rich regions up to 100 mM NaCl, while it removed them from GC-rich ones above 40 mM NaCl. We attempted to explain this intriguing property by fluorescence resonance energy transfer (FRET). Besides, tTALE-FP shared the advantages that other FP-DBPs had, such as no photo-cleavage or no structural deformation, and reversible staining^25^. Finally, we demonstrated the power of the tTALE-FP for the visualization of real-time restriction enzyme digestion reactions on single-molecule DNA by comparing it with the YOYO-1 stained DNA molecule.

## Results and Discussion

### Truncated transcription-activator-like effectors - fluorescent proteins (tTALE-FP)

**Figure 1:**
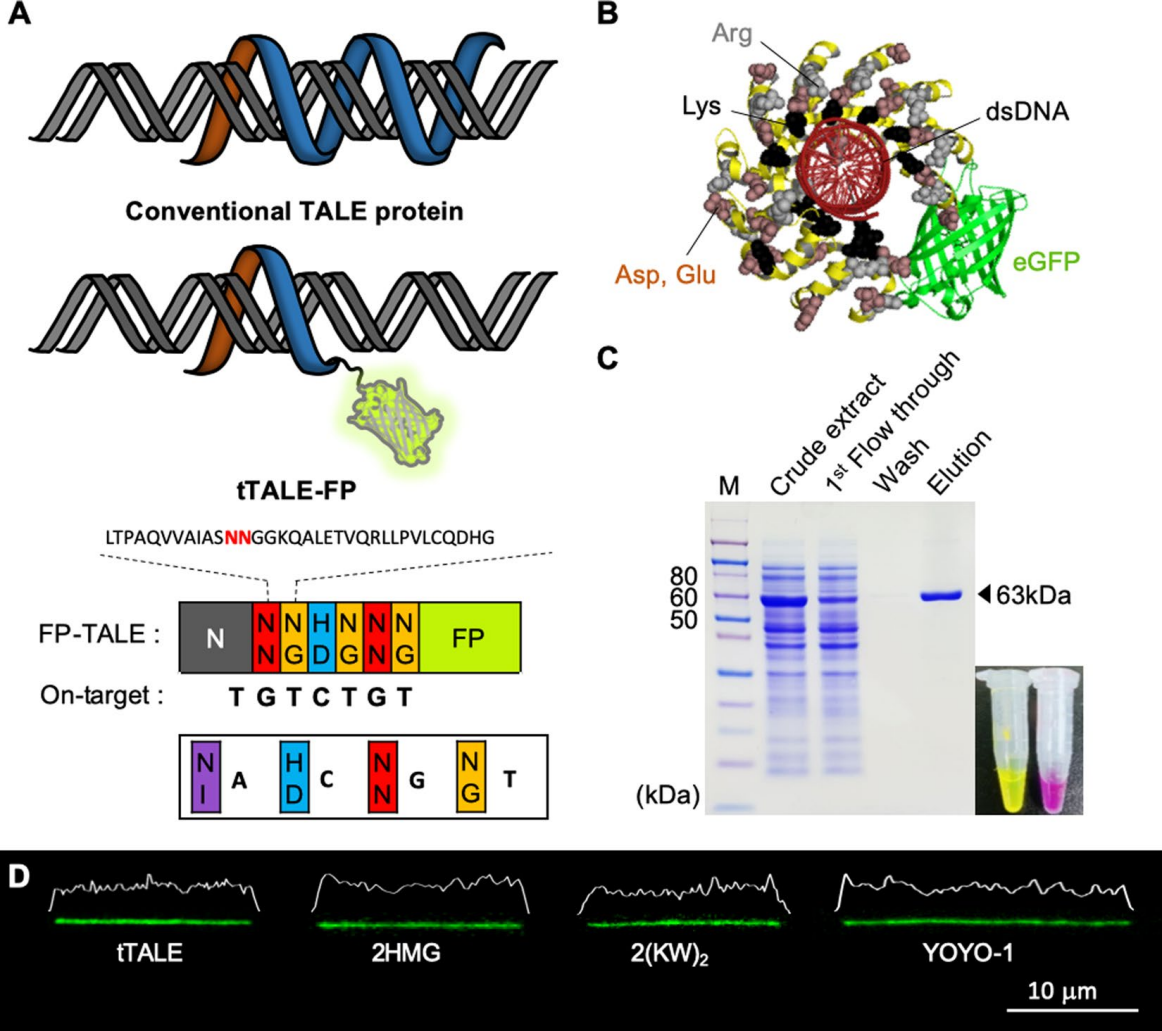
Truncated TALE-FP. (**A**) Illustration of conventional TALE on DNA and binding domains designed to recognize 7-mer ‘TGTCTGT’ fused with FP. (**B**) The combined structure of tTALE-FP and DNA. (**C**) SDS-PAGE for affinity chromatography purification of TALE-eGFP (63 kDa). Purified tTALE-eGFP and tTALE-mCherry. (**D**) Fluorescence microscopic images and intensity profiles for tTALE-eGFP (2 nM) stained λ DNA molecule (48.5 kb) compared with 2HMG-eGFP^34^, 2(KW)_2_-eGFP^25^, and YOYO-1 stained ones.

Figure 1 illustrated a novel FP-DBP constructed from the gene of a transcription activator-like effector nuclease for a 20-mer target sequence (TGTCTGTGGCCTGGTGCCTG)^41^. TALE has three domains: N-terminal, DNA binding repeat, and C-terminal^42^. The N-terminal consists of 133 amino acids that bind thymine^43^. We added an FP gene at the C-terminus of 7-mer (TGTCTGT) DNA binding domain gene. A reason to make 7-mer tTALE-FP was that there are eight target sites in the λ DNA, while there are two sites for 8-mer, one for 9-mer, and none for 10-mer or larger. We did not include TALE’s C-terminal domain because it contains a transcriptional activation domain and a nuclear localization signal^41^. Each DNA binding repeat domain comprises 34 or 35 amino acids, and the 12th and 13th amino acids per repeat, which determine the base specificity, called repeat variable di-residues (RVDs) (Fig. 1A)^44^. On the other hand, it is controversial to predict an RVD’s binding base. According to Boch *et al*., the NN in TALE RVD has a similar preference for G and A^45^. However, Miller *et al*. reported a further study that the NN has a higher binding affinity for G than A^46^. Moreover, they proved that neighboring repeats also influence specificity. Their result suggests that NG-NN-NG has a base specificity for T-G-T. Therefore, we used TGTCTGT as the target sequence in the remaining part of this paper (Fig. 1A).

TALE’s combined structure is a right-handed superhelix that wraps around the double-stranded DNA molecule^47^. Figure 1B shows a molecular model that predicts three-quarter wrapping of the double helix, which we generated by using PyMol from a combination of data for TALE^48^, eGFP^49^, and a complex of TALE and DNA^47^. The predicted structure shows lysine residues primarily in the inside of the superhelix. They may contribute to the electrostatic interaction with the DNA phosphate backbone. The outside structure seems neutral or slightly negatively charged due to the combination of arginine, aspartate, and glutamate, which implies that its protein surface interacts weakly with negatively charged DNA backbones.

We prepared tTALE-eGFP and tTALE-mCherry (Fig. 1C). Figure 1D demonstrated tTALE-FP stained λ DNA molecules in 1 × TE buffer (10 mM Tris, 1 mM EDTA, pH 8.0), compared with other staining reagents, such as 2(KWKWKKA)-eGFP^25^, 2HMG-eGFP^34^, and YOYO-1. For these images, we elongated DNA molecules by a droplet-spreading protocol in which a drop of DNA solution is placed on a positively-charged coverslip. A subsequent quick covering with a second glass slide then induces a local flow, which results in the DNA collapsing to the surface in an extended conformation. The average extension of the tTALE-FP stained λ DNA molecule was with a length of 15 µm similar to other FP-DBPs (2HMG-eGFP and 2(KWKWKKA)-eGFP), while the YOYO-1 stained DNA showed a greatly increased length of 21.5 µm. Given that the expected length of a fully-extended λ DNA is about 16.3 µm, this demonstrates that tTALE-FP does not significantly distort the native DNA structure, whereas YOYO-1 binding induces significant lengthening^50^.

### Salt-dependence of tTALE-FP stained λ DNA molecules

**Figure 2:**
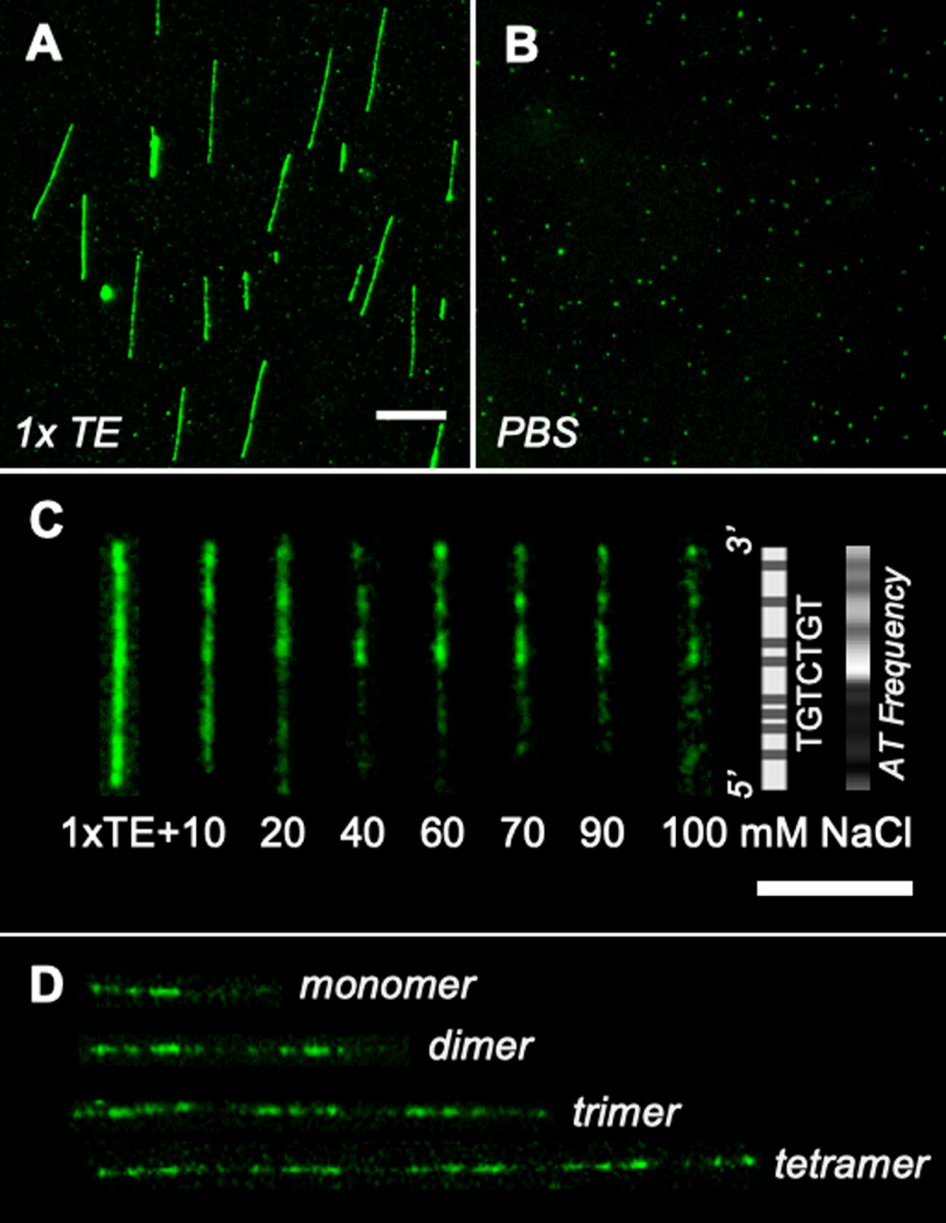
Salt-dependence of tTALE-FP staining. (**A**) 1 × TE (Tris-EDTA: 10 mM Tris, 1 mM EDTA, pH 8.0). (**B**) 1 × PBS (phosphate-buffered saline: 137 mM NaCl, 2.7 mM KCl, 10 mM Na_2_HPO_4_, 1.8 mM KH_2_PO_4_, pH 7.4). (**C**) λ DNA molecules after the addition of NaCl (10–100 mM) to 1 × TE buffer. Sequence-specific *in silico* maps of TGTCTGT and AT frequency. Scale bar = 10 μm. (**D**) λ concatemers in 40 mM NaCl added 1 × TE (i.e., tetramer = 4 × 48.5 kb = 194 kb). All DNA molecules are stained by preincubation in 2 nM tTALE-FP.

The primary motivation of this study is to develop a salt-tolerant FP-DBP. Thus, we compared the two extreme cases of low and high-salt concentrations, such as 1 × TE and PBS. 1 × TE buffer has 10 mM Tris and 1 mM EDTA. Since Tris-H is a weak acid, the ionic strength should be less than 10 mM at pH 8.0 by considering their p*K*a values. Previously, we determined 5.26 mM as the ionic strength in a 1 × TE buffer solution^50^. On the other hand, phosphate-buffered saline (PBS) contains a high concentration of salts (137 mM NaCl, 2.7 mM KCl, 10 mM Na_2_HPO_4_, 1.8 mM KH_2_PO_4_). In contrast to Fig. 2A (1 × TE), Fig. 2B (1 × PBS) shows no DNA molecules except scattered spots. Next, we gradually increased NaCl concentrations into 1 × TE buffer, which revealed characteristic patterns for λ DNA molecule ranging from 40 mM to 100 mM (Fig. 2C). For larger than 100 mM NaCl, images were similar to the case of 1 × PBS buffer.

Interestingly, λ DNA images from 40 mM to 100 mM have consistent patterns that seem related to the A/T frequency *in silico* map (Fig. 2C). This pattern is similar to previous results of AT-specific polypyrrole compound^6^ and FP-DBPs of H-NS and HMG^11^. This pattern is even retained in concatenated λ-DNA molecules, which allows us to readily identify the number of monomers and their orientation (Fig. 2D). However, it required further characterization of why tTALE-FP generated the AT-specific pattern. We note that tTALE-FP has been designed to show preferred binding to seven nucleotides target sequence (TGTCTGT). While this does not match with the observed staining pattern shown in Fig. 2C, we cannot conclude that therefore, target binding is absent. Instead, it just means that the overall DNA affinity of the protein might be too high to resolve this binding. To be more quantitative, we designed a bulk assay to measure tTALE-FP’s binding affinity to target, AT-rich, and GC-rich sequences, respectively.

### The binding affinity of tTALE-eGFP by FRET assay and single-molecule assay

**Figure 3:**
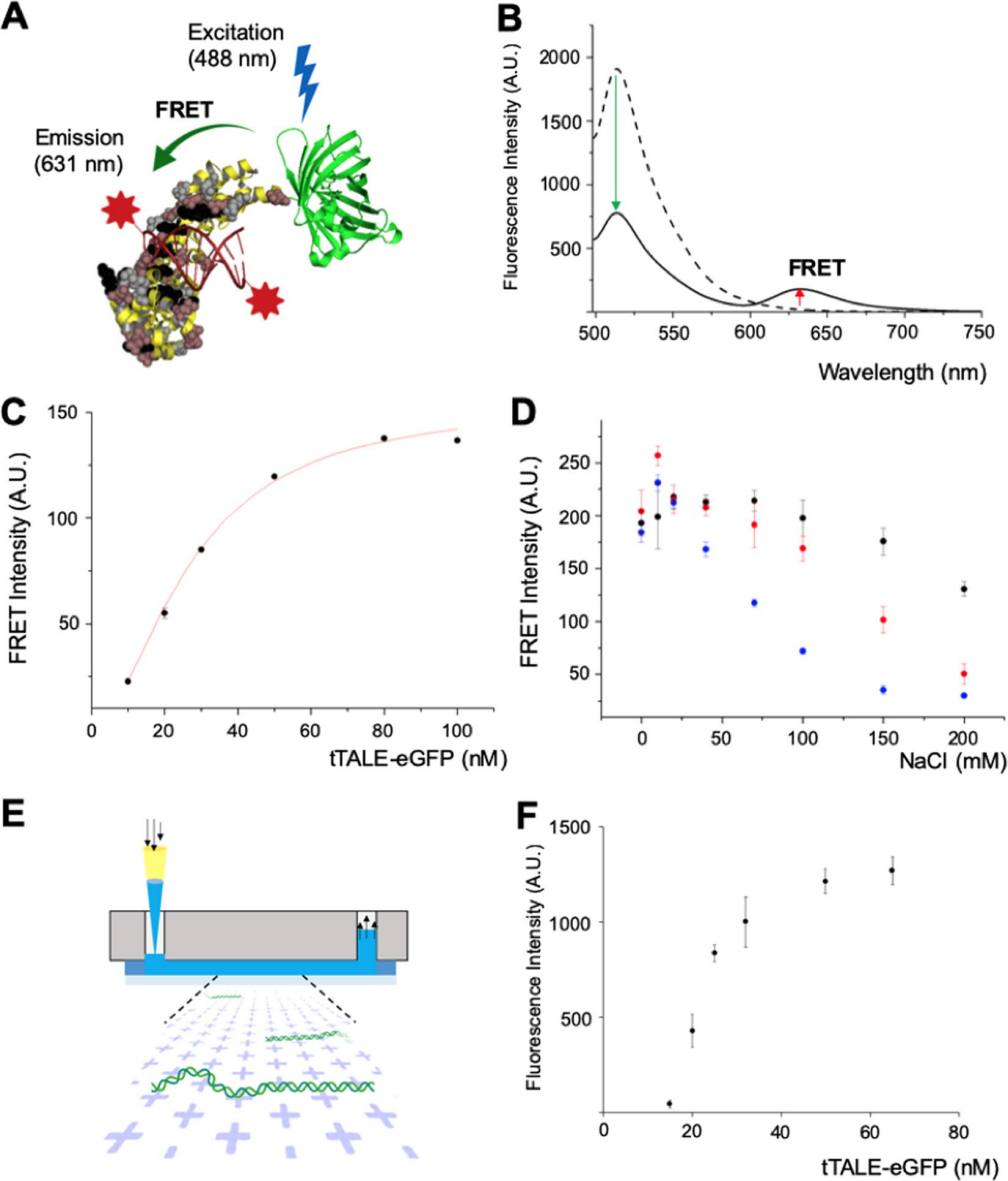
Quantitative analysis with FRET and flow cell with surface-immobilization for the binding affinity of tTALE-eGFP on DNA molecules. (**A**) Schematics of FRET between tTALE-eGFP and ATTO-590 labeled DNA molecules. (**B**) Emission spectra from the 30 nM tTALE-eGFP only (dotted line) and the 30/100 nM complex of tTALE-eGFP/5′-ATTO-590 labeled-target sequence (solid line). (**C**) Fluorescence intensity measurement of tTALE-FP with 1 pM 5′-ATTO-590 labeled-target dsDNA oligomer at 631 nm. *K*_d_ = 26.7 nM. (**D**) Change of fluorescence intensity of 30/100 nM tTALE-eGFP/5′-ATTO-590 labeled dsDNA oligomers at 631 nm with NaCl concentration. •: target DNA. : AT-rich DNA. : GC-rich DNA. (**E**) Schematics of the flow cell with surface-immobilization (made in ©BioRender - biorender.com). (**F**) tTALE-eGFP concentration-dependent fluorescence intensities of λ DNA. (*see* Materials and Methods for more details).

We designed a fluorescence resonance energy transfer (FRET) assay to measure the binding affinity of tTALE-eGFP to double-stranded DNA oligomers labeled with ATTO-590, which followed our previous paper^51^. Fig. 3A illustrates how a significant FRET emission occurs between tTALE-FP and DNA. Figure 3B presents a typical intensity changes: the increase of ATTO-590 emission at 630 nm and the decrease of tTALE-eGFP emission at 510 nm. To characterize sequence-specificity, we prepared three types of ATTO-590 labeled DNA oligomers: TGTCTGTATG as the target sequence, GAAGAAAATGATCTA as AT-rich one, and GCGACCTCGCGGGTT as GC-rich one. We designed AT-rich and GC-rich sequences from the λ genome. First, the FRET assay allowed us to measure the dissociation constant (*K*_d_ = 26.7 nM) of tTALE-eGFP and the target sequence in 1 × TE (Fig. 3C). This value is much lower than other FP-DBPs such as 14.6 µM for 2(KW)_2_-eGFP^25^, and 586 nM for 2HMG-eGFP^34^. Fig. 3D demonstrates that the increase of NaCl concentrations reduces FRET intensities with different decreasing trends for three DNA oligomers. More specifically, for 20 mM or lower NaCl concentrations, the data from three oligomers are similar within the error range in an unsaturated protein condition (tTALE-FP: 30 nM). From 40 mM to 150 mM NaCl, GC-rich data are significantly lower than the others, which implies that the GC-rich sequence has a weak affinity for tTALE-FP. This result explains AT-specific patterns of λ DNA that we observed in Fig. 2B. Notably, the target sequence shows higher FRET efficiency than the others from 150 mM to 200 mM.

Besides, we conducted a single-molecule assay by using tTALE-eGFP stained λ DNA (Fig. 3E). First, we loaded unstained λ DNA solution on a positively charged surface within a flow cell and flushed tTALE-eGFP of given concentrations (2 ~ 64 nM). Then we flushed out unbound FP with 10 µL of 1 × TE buffer. We analyzed the integrated intensity of individual molecules using a lab-made python program. Figure 3F shows a typical protein-DNA binding curve based on Hill’s equation. Nevertheless, it is challenging to define *K*_d_ because of unknown the number of FP on DNA (*n*).

1 $${K}_{d}=\frac{[DNA]{[protein]}^{n}}{[DNA-n\times protein]}$$

Roughly, it is possible to assume *n* = 1 and [DNA] as the concentration of binding sites, which suggests that a protein concentration corresponds to *K*_d_ when the intensity equal to half of the maximum intensity. This assumption predicts *K*_d_ = 22 nM from Fig. 3F, which agrees roughly with *K*_d_ = 26.7 nM that we measured in the FRET bulk assay. However, we found that surface-immobilized DNA molecules were not efficient for staining and destaining. Therefore, we could not see DNA with 8 nM or lower tTALE-eGFP, while we used 2 nM for other preincubation results. Moreover, we observed randomly destained patterns instead of the AT-specific pattern when we flushed the flow cell with a NaCl solution. Therefore, for the next experiment, we preincubated DNA with tTALE-FP in a test-tube before loading into a positively-charged surface.

### Surface-immobilized tTALE-FP stained DNA with the AT-specific pattern

**Figure 4:**
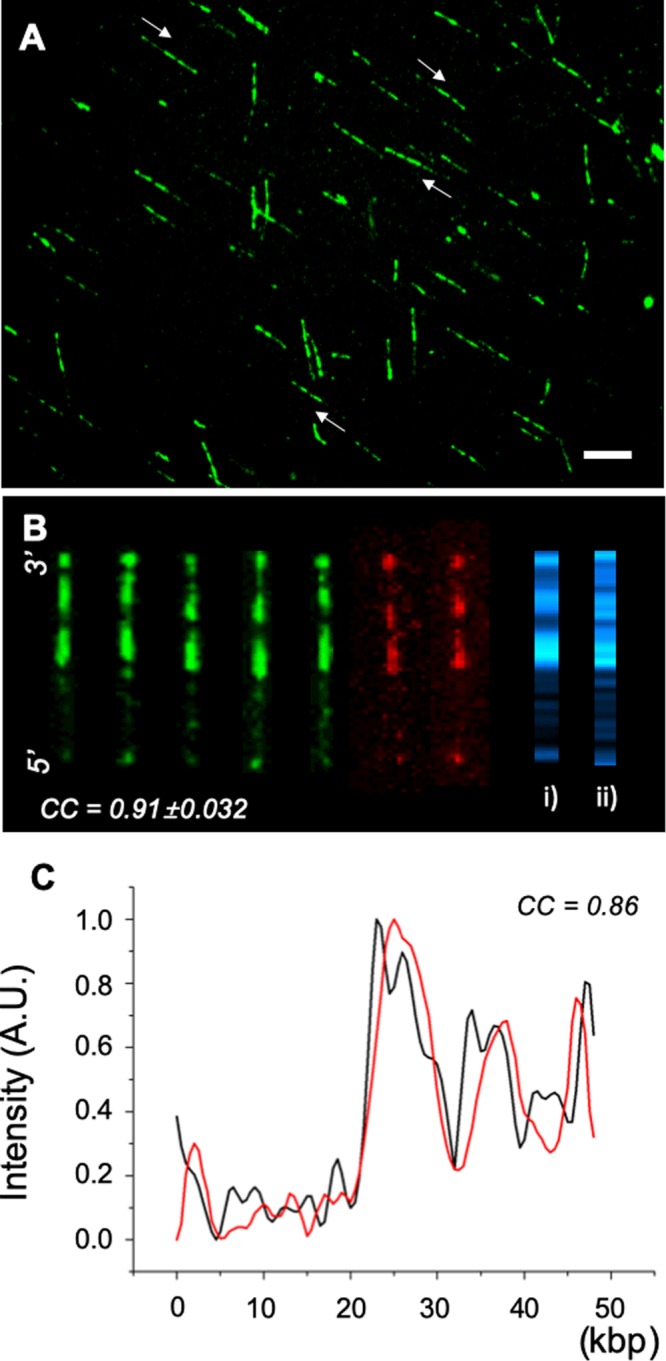
tTALE-FP stained λ DNA molecules immobilized on a positively charged surface in 70 mM NaCl. (**A**) Fluorescence micrographs of surface-immobilized AT-specific patterned DNA molecules with stained by preincubation in 2 nM tTALE-FP without flushing unbound tTALE-FP. The white arrows indicate the molecular direction of λ DNA (scale bar = 10 μm). (**B**) Aligned λ DNA, eGFP (green) and mCherry (red). *cc* = 0.91 ± 0.032 among molecules. (*i*) Consensus map. (*ii*) AT-frequency *in silico* map. (**C**) Comparison of consensus map (red) and AT-frequency *in silico* map (black). *cc* = 0.86.

Figure 4A shows 2 nM tTALE-FP stained λ DNA molecules immobilized on a positively charged surface after preincubation in a 70 mM NaCl concentration. This AT-specific staining is useful because it can tell the orientation of the λ genome, as indicated by the white arrows. To quantitatively evaluate the consistency of the tTALE-FP DNA staining, we calculated the Pearson cross-correlation coefficient (*cc*) among molecular images using a lab-made Python program, which was available on the website for our previous paper^6^. The *cc* value was 0.91 ± 0.032, which suggests that molecular patterns are consistent and reproducible (Fig. 4B).

Alternatively, we made the consensus intensity pattern by combining molecular intensity profiles (*i*), which were compared with *in silico* AT-frequency map for λ DNA molecules (*ii*). Figure 4C compares the fluorescence intensity profiles for (*i*) and (*ii*) with *cc* = 0.86. This comparison for the two intensity graphs demonstrates that tTALE-FP produces DNA staining patterns, which show a good correlation with AT-rich sequences, even though it cannot resolve highly detailed sequence features.

### Flow-cell tethered DNA molecules reversibly stained with tTALE-FP

**Figure 5:**
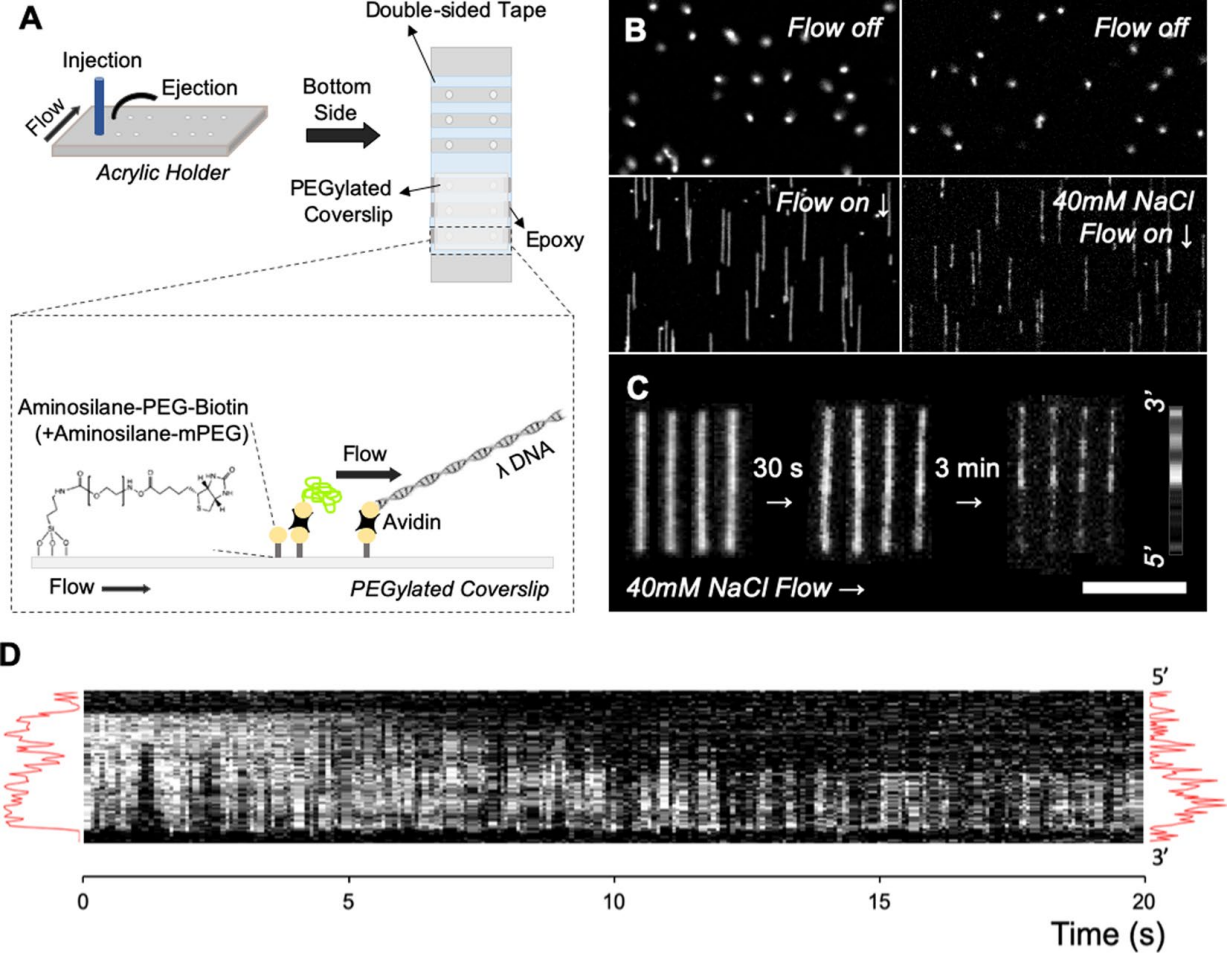
Tethered λ DNA reversibly stained with tTALE-FP in a flow chamber. (**A**) Schematics of DNA tethering in a flow cell. (**B**) Fluorescence microscopic images of tethered λ DNA molecules with mushroom-like coiled conformation (flow-off) and elongated one (flow-on). The experiment steps were DNA tethering, 2 nM tTALE-FP loading, and flushing with 1 × TE or 40 mM NaCl added solutions. (**C**) Time-lapse images following the addition of 40 mM NaCl solution to reveal the AT-specific pattern in a flow cell with the consensus pattern from Fig. 4B. Scale bar = 10 μm. (**D**) The kymogram represents a 20-sec destaining process of tTALE-FP from λ DNA in 70 mM NaCl solution (*see* Movie S1).

Figure 5 demonstrates the application of tTALE-FP to tethered DNA via biotin-avidin interaction in a flow cell^35^. The approach of surface-tethered DNA elongated by a flow has advantages over surface-immobilization because there is only a single attachment point. In contrast, surface-immobilization will result in many contact points and thus could significantly affect protein activity, as shown in Fig. 3E,F ^52^. Moreover, this approach does not need preincubation in a test tube. We tethered DNA molecules first, loaded tTALE-FP, and then flushed with 1 × TE solution to elongate DNA. The elongation length of the tethered tTALE-FP-stained λ DNA in the flow cell was 13.44 ± 0.38 µm, representing 83% of the full contour length of native λ DNA (16.3 µm = 0.337 nm × 48,502 bp), at a flow rate of 100 μL/min. This length is consistent with other FP-DBP results that range from 13.4 μm (82%) to 14.6 μm (89%) as well as our previous simulation result that predicted 83% in stretching the same flow cell^34^.

Figure 5C,D present the destaining process in a salt solution. 40 mM NaCl added 1 × TE buffer solution gradually revealed AT-specific patterns, which agreed with the surface-immobilized DNA pattern in Fig. 4B. More specifically, the tTALE-FP staining of the λ DNA molecules were homogeneous in the beginning, and after 30 seconds of adding a 40 mM NaCl solution into the flow cell, these fluorescent proteins started to fall off and revealed the AT-specific pattern in three minutes. We attempted to make a movie for Fig. 5C, but it was challenging because a continuous illumination for three minutes caused severe bleaching on DNA images. In order to reduce the effects of bleaching, we sped up the destaining process by switching to a buffer containing 70 mM NaCl. Therefore, we enhanced NaCl concentration to 70 mM to destain quickly for 20 seconds without bleaching (Fig. 5D). Although the pattern does not agree perfectly well with the AT-specific intensity profile, this kymogram demonstrates the destaining process in the GC-rich region (5′).

### Real-time observation of tTALE-FP staining

**Figure 6:**
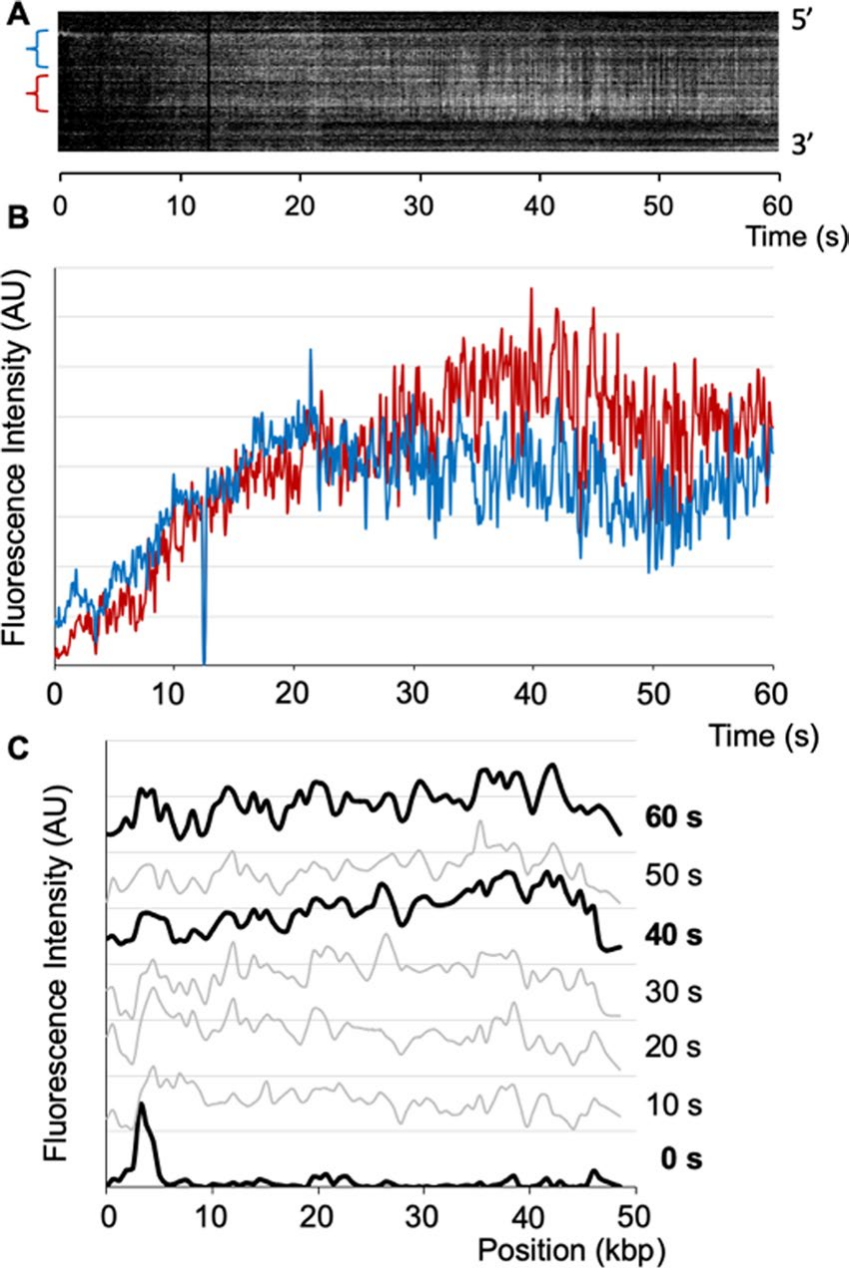
Real-time observation of λ DNA staining with tTALE-FP. (**A**) Kymogram of λ DNA staining with tTALE-FP (2 nM) in a 1 × TE buffer. Each blue and red bracket represents the GC-rich and the AT-rich regions, respectively. (**B**) Fluorescence intensity profiles over time for the GC-rich (blue) and the AT-rich (red) regions of tTALE-FP stained λ DNA. (**C**) Fluorescence intensity along λ DNA (5′ → 3′) at each 10-sec interval (*see* Movie S2).

Next, we attempted to observe the tTALE-FP staining process in real-time. For this purpose, we first stained tethered λ DNA with tTALE-FP to set up the focal position in the microscope. Then, we removed tTALE-FP with 150 mM NaCl added 1 × TE and subsequent washing with a 1 × TE buffer solution. After a while, we loaded 2 nM tTALE-FP in 1 × TE. Figure 6A presents a kymogram of the staining process for 60 seconds. Figure 6B shows fluorescent intensity profiles from the kymogram to show two intensity changes for AT-rich (red) and GC-rich (blue) regions over time. For the first 20 seconds, staining increased with similar rates for both, but the AT-rich sequence was stained preferentially over the GC-rich one from 20 to 50 seconds. However, intensity gaps between the two parts became narrowed at 60 seconds. Figure 6C illustrates the change of fluorescent intensity profiles along the genome (5′ → 3′). The 40-second profile shows that the GC-rich region (5′) has a lower intensity than the AT-rich one (3′), but the 60-second intensity profile shows that the staining becomes more uniform along the λ genome.

### Real-time monitoring of restriction-enzyme reaction with tTALE-FP vs. YOYO-1

**Figure 7:**
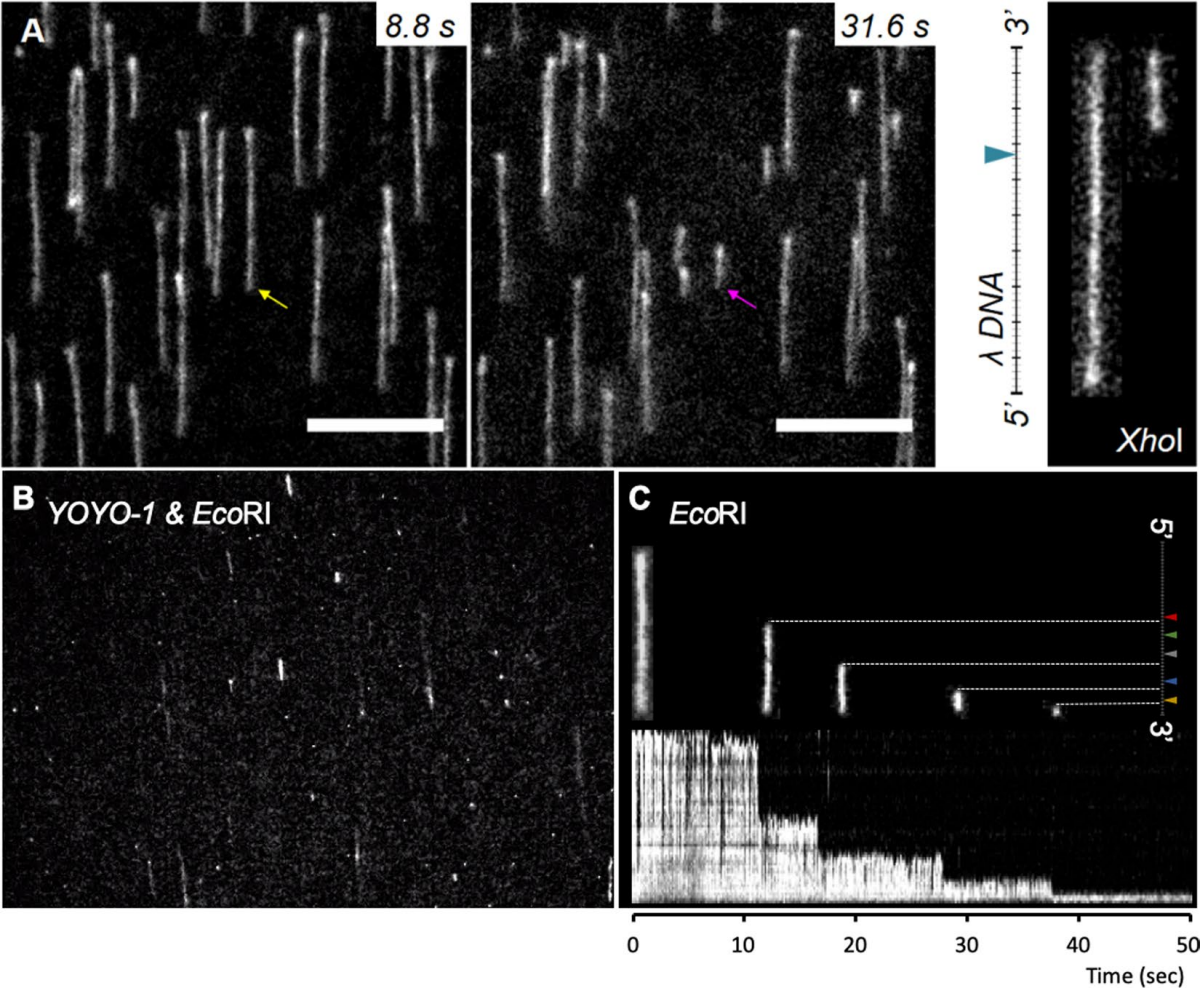
Real-time monitoring of restriction enzyme reaction with tTALE-FP and YOYO-1. (**A**) *Xho*I digestion of tTALE-FP (2 nM) stained λ DNA (Scale bars = 10 μm) (*see* Movie S3). (**B**) *Eco*RI digestion of YOYO-1 stained DNA as a control (*see* Movie S4). (**C**) Kymogram of tTALE-FP stained λ DNA digested by *Eco*RI: four consecutive cutting of a single λ DNA (*see* Movie S5). Five DNA images represent a DNA molecule cut by *Eco*RI at four out of five target sites.

Figure 7 illustrates real-time monitoring of restriction enzyme reactions on tethered DNA in a flow cell (*see SI* Movies S3, S4, and S5). On the fully stretched λ DNA, the *Xho*I enzyme (CTCGAG), a single cutter of λ DNA (33.5 kb and 15.0 kb), created digested fragments that move through the microscopic view in the movie (*see* SI Movie S3). After 31.6 seconds, there were many digested DNA fragments of 15 kb (3.8 μm) tethered on the surface (Fig. 7A).

As a comparison, we performed an *Eco*RI restriction enzyme reaction using YOYO-1 stained DNA (Fig. 7B and *SI* Movie S4). Right after introducing the enzyme, most DNA molecules entirely disappeared instead of sequence-specific digestions. A couple of previous studies reported the inhibition of TOTO-1 and YOYO-1 against endonuclease activity to cut DNA^29,30^. There was another study to visualize endonuclease digestion of TOTO-1 stained DNA in a nanochannel, in which they reduced TOTO-1 concentration down to 1 dye: 20 bp while 1 dye: 4 bp is a typical concentration^53^. Therefore, we could not expect proper digestion by YOYO-1stained DNA, but Fig. 7B was still an unexpected result. As a control, we applied restriction enzyme digestion buffer (NEB CutSmart) to YOYO-1 stained λ DNA without *Eco*RI. Although there was some photo-induced DNA cleavage, it was quite different from the case with the enzyme (Fig. 7B).

In contrast, the *Eco*RI enzyme safely digested tTALE-stained λ DNA, as shown in Fig. 7C (*SI* Movie S5). The kymogram shows enzymatic digestion process. A single λ DNA was cut four times out of five recognition sites. It is worthwhile to compare Movie S4 (YOYO-1) and Movie S5 (tTALE-FP). Both movies used *Eco*RI, which should generate 3.5 kb (3.5 kbp × 0.34 nm = 1.2 µm) of tethered DNA after full digestion. The last image of Movie S5 shows a bunch of 3.5 kb DNA molecules, but that of Movie S4 does not show them, which implies that there was not proper digestion of YOYO-1 stained DNA. In conclusion, this comparison demonstrates the use of tTALE-FP staining for monitoring enzymatic reactions.

From the molecular images of Fig. 7C, it is notable that there are consistent discrepancies between the expected and observed sites. This phenomenon origins from the fact that the smaller fragments stretched less than large ones compared with the expected lengths. A previous study explained this shortening for tethered DNA at one end in a shear flow^54^. DNA stretch (*X/L*) reduces with the molecular size (*N*), as shown in Eq. (2) ^55^. Therefore, the smaller DNA makes the stretch (*X/L*) shorter with the same flow with the constant *C*.

2 $$X/L=1-C/{N}^{5/9}$$

### Restriction-enzyme reaction confirmed by AT-specific map

**Figure 8:**
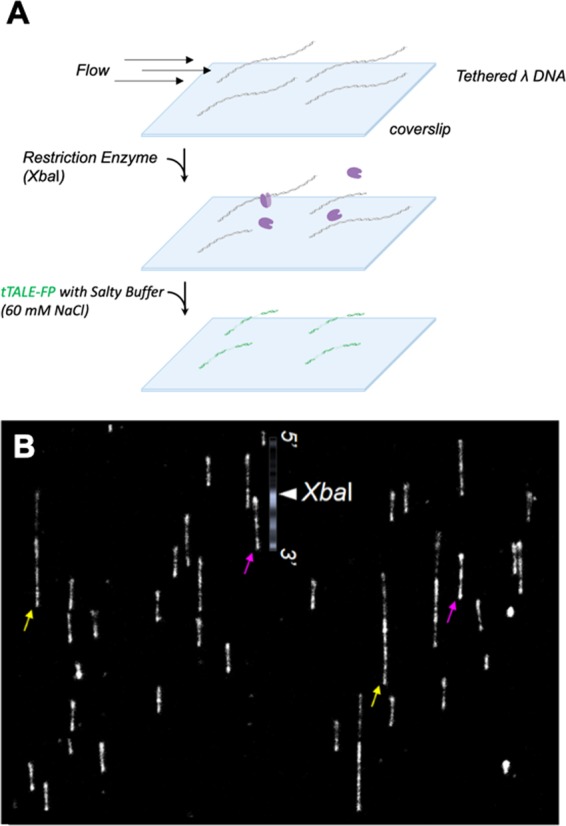
DNA digestion and confirmation by the optical map. (**A**) Schematic of *Xba*I digested λ DNA patterned with tTALE-FP (2 nM) to reveal the genome-specific pattern (made in ©BioRender - biorender.com). (**B**) λ DNA in a mixture of tTALE-FP and NaCl solution. Yellow arrows indicate whole λ DNA and pink arrows indicate digested λ DNA, compared with AT-specific consensus genomic map.

Finally, we combined DNA digestion and post-staining with a NaCl buffer, as shown in Fig. 8. After the *Xba*I reaction, fresh 2 nM tTALE-FP mixed with 60 mM NaCl solution flowed through the flow cell. Figure 8B illustrates sequence-specifically labeled DNA, which allowed us to confirm the site of the restriction enzyme digestion by comparing the result with the AT-specific consensus λ genome pattern that we obtained in Fig. 4B.

## Conclusion

In this study, we developed tTALE-FP as a salt-tolerant FP-DBP. Unlike other FP-DBPs, tTALE-FP is compatible with staining at elevated salt concentrations (40–100 mM NaCl), depending on the AT-content to which it binds preferentially. On the other hand, its preference for binding AT-rich regions can be helpful in certain conditions, e.g., to determine the DNA orientation. We also demonstrate that its DNA binding interferes little with the activity of DNA-processing proteins such as DNA restriction enzymes. The likely reason is that it seems to not substantially alter the DNA conformation, as opposed to, e.g., intercalators such as YOYO-1. Finally, it has an additional advantage over intercalators in that it does not induce photo-nicking or photo-cleavage of the DNA.

## Material and Methods

### Chemicals

All DNA primers were purchased from Cosmogenetech (Seoul, Korea). The DNA ladder and biotin-labeled DNA oligomer were purchased from Bioneer (Daejeon, Korea). The λ DNA (48.5 kb) and all enzymes were purchased from New England Biolabs (Ipswich, MA). T4 GT7 DNA (165,644 bp) was purchased from Nippon Gene (Tokyo, Japan). The TALEN plasmids (*RHD*_Exon4_TALEN_L) were obtained from Yonsei University College of Medicine (Seoul, Korea). The *AccuPower® Pfu* PCR PreMix kit was purchased from Bioneer (Daejeon, Korea). The *E*. *coli* strains DH5α and BL21 (DE3) were purchased from Yeastern (Taipei, Taiwan). Epoxy was obtained from Devcon (Riviera Beach, FL). N-trimethoxymethyl silyl propyl-N,N,N-trimethyl ammonium chloride in 50% methanol was purchased from Gelest (Morrisville, PA), and N-[3-(trimethoxysilyl)propyl]ethylene diamine was purchased from Sigma (St. Louis, MO). mPEG–succinimidyl valerate and biotin–PEG–succinimidyl carbonate (both MW 5000) were purchased from Laysan Bio (Arab, AL). Ni-NTA agarose resin and the disposable empty gravity column were purchased from Qiagen (Venlo, Netherlands). All other chemicals were obtained from Sigma-Aldrich (St. Louis, MO).

### Microscopy

The microscopy system comprised an inverted microscope (Olympus IX70, Tokyo, Japan) equipped with 60× and 100× Olympus UPlanSApo oil immersion objectives and an illuminated LED light source (SOLA SM II light engine, Lumencor, Beaverton, OR). The light passed through the corresponding filter sets (Semrock, Rochester, NY) for excitation and emission of light. Fluorescence images were captured using a scientific complementary metal–oxide–semiconductor (sCMOS) camera (PRIME; Photometrics, AZ, USA) with 100 ms exposure time and images were stored as 16-bit TIFF file using Micro-manager. ImageJ was utilized for image processing using a Java plug-in developed in our laboratory for image analysis. Python programs were used to compare the fluorescence intensity of two DNA images.

### tTALE-FP construction, purification, and cell growth

The plasmid pET-15b (Novagen, Germany) was used to construct the recombinants and express tTALE-FP. The fluorescent protein part and TALE part were amplified and fused by a linker sequence (5′-CTT GTA CAG CTC GTC CAT GCC-3′) through a typical overlap PCR process. For the fluorescent protein (EGFP) part, a forward primer (5′- GGC GGC TCT GGC GGC ATG GTG AGC AAG GGC GAG G -3′) and reverse primer (5′- ATT TCA GGA TCC TTA CGC CTT GTA CAG CTC GTC CAT G -3′) were used in 20 μL of *Pfu* PCR PreMix supplemented with 0.25 mM of each dNTP and 1.5 mM MgCl_2_. For the TALE part, the TALEN plasmid was isolated from *RHD*_Exon4_TALEN_L^41^. A forward primer (5′- ATT TCA CAT ATG GAT CTA CGC ACG CTC GGC TAC -3′) and reverse primer (5′- CAC CAT GCC GCC AGA GCC GCC TTC ACT TTT GAC TAG CAA CGC GGC -3′) were used as described above. The fluorescent protein part and the TALE part were fused by the mediating linker site. This PCR process was performed by incubation at 95 °C for 2 min, 94 °C for 1 min, 55 °C for 1 min, and 72 °C for approximately 4 min for 12 cycles. After constructing the tTALE-FP templates, an overlap PCR was performed with a forward primer (5′- ATT TCA CAT ATG GAT CTA CGC ACG CTC -3′) and reverse primer (5′- ATT TCA GGA TCC TTA CGC CTT GTA CAG CTC -3′, N′-*tTALE-FP*-C′). Except for the tTALE-FP template construction step, all PCR analyses were performed by incubation at 95 °C for 2 min, 94 °C for 1 min, 55 °C for 1 min and 72 °C for approximately 4 min for 35 cycles. After the purification of the PCR products, NdeI and BamHI were used for the fragment insertion into the pET-15b vector. The ligation reaction was performed for 1 hr at 16 °C.

The recombinant tTALE-FP plasmids were transformed into *E*. *coli* DH5α strains using the heat shock method, and the DH5α transformation was assessed by colony PCR. For the protein over-expression, the plasmids were purified and transformed into the *E*. *coli* BL21 (DE3) strains. The transformed cells were recovered in fresh LB media without ampicillin for 1 hr and spread evenly on an LB agar plate with ampicillin overnight. A single colony was incubated in fresh LB medium supplemented with ampicillin for 12 hr. After saturation, the transformed cells were cultured to an optical density of approximately 0.8 at 37 °C with corresponding antibiotics. The tTALE-FP protein over-expression was induced by a final concentration of 1 mM IPTG overnight on a shaker at 20 °C and 150 rpm. The cells were lysed by ultrasonication for 30 min, and the cell debris was centrifuged at 13,000 rpm for 10 min at 4 °C. Then, 6× His-tagged tTALE-FP was purified using affinity chromatography with Ni-NTA agarose resin. The mixture of cell proteins and resin was kept on a shaking platform at 4 °C for 1 hr. The lysate containing proteins bound to Ni-NTA agarose resin was loaded onto the affinity column, washed with protein wash buffer (50 mM Na_2_HPO_4_, 300 mM NaCl, 20 mM imidazole, pH 8.0) several times, and finally eluted with protein elution buffer (50 mM Na_2_HPO_4_, 300 mM NaCl, 250 mM imidazole, pH 8.0). After the SDS-PAGE analysis, further protein purification processes were not performed (Fig. 1C).

### Positively charged glass surface preparation

Glass coverslips (22 × 22 mm) were stacked in a Teflon rack, immersed in a piranha etching solution (30:70 v/v H_2_O_2_/H_2_SO_4_) for 3 hr and rinsed thoroughly with deionized water. After sonicating the neutralized coverslips for 30 min, two types of derivatized surfaces could be created. Then, 400 μL of N-trimethoxymethyl silylpropyl-N,N,N-trimethylammonium chloride in 50% methanol were added to 250 mL of deionized water. The glass coverslips were incubated in this solution for 16 hr at 65 °C, followed by rinsing with ethanol to make a positively charged glass surface. These coverslips were used within two weeks of their preparation.

### Single DNA molecule imaging

To image the tTALE-FP-stained DNA, the DNA molecules (approximately 15 ng/µL) were mixed with tTALE-FP (2–20 nM) in 1 × TE (10 mM Tris, 1 mM EDTA, pH 8.0). NaCl salt buffer was prepared by diluting 150 mM NaCl in 1 × TE buffer for each concentration. The mixture was incubated at room temperature for 10 min and then added to a positively charged surface. This coverslip was mounted on a glass slide. After the sample preparation, the DNA molecules were imaged under a microscope.

### Fluorescent resonance energy transfer (FRET)

Three kinds of 5′-ATTO^TM^ 590-labeled double-stranded DNA (dsDNA) were prepared by hybridizing 5′-ATTO^TM^ 590-labeled single-stranded DNA (ssDNA) and its complementary ssDNA (target DNA: TGTCTGTATG, A/T rich DNA: GAAGAAAATGATCTA, G/C rich DNA: GCGACCTCGCGGGTT). 10 µL of every complementary ssDNA molecules (100 µM) was mixed with 20 µL of 100 mM NaCl. Each mixture was heated at 95 °C and cooled gradually (0.5 °C/min) to 20 °C and stored at 12 °C to form a duplex. The 5′-ATTO^TM^ 590-labeled dsDNA (100 nM) was incubated with tTALE-eGFP (300 nM) in TE (pH 8.0), and fluorescence was scanned from 498 to 750 nm with excitation at 488 nm. To measure *K*_d_, the 5′-ATTO^TM^ 590-labeled dsDNA (100 nM) containing target sequence was titrated with tTALE-eGFP (10, 20, 30, 50, 80 and 100 nM), and fluorescence was measured at 631 nm with excitation at 488 nm. To investigate the change of fluorescence intensity at 631 nm with corresponding NaCl concentration, three different dsDNA (100 nM) and tTALE-eGFP (30 nM) were incubated separately with different NaCl solutions (0, 10, 20, 40, 70, 100, 150, 200 mM), and fluorescence was measured at 631 nm with excitation at 488 nm.

### PEGylated glass surface preparation

To obtain surfaces coated with primary amine groups, 2 mL of N-[3-(trimethoxysilyl)propyl] ethylenediamine and 10 mL of glacial acetic acid were added to 200 mL of methanol. The glass coverslips were incubated in this solution for 30 min, sonicated for 15 min, incubated again for 16 hr at room temperature, and rinsed with methanol and ethanol. The aminosilanized glass was coated with PEG. Then, 80 mg of mPEG–succinimidyl valerate and 2 mg of biotin–PEG–succinimidyl carbonate were added to freshly prepared 0.1 M sodium bicarbonate. This solution was completely mixed and briefly centrifuged. In total, 50 μL of PEG solution was added to a clean slide glass without air bubbles, covered with aminosilanized glass overnight, and rinsed carefully with water.

### DNA-tethering in a flow cell

The flow chamber comprised an acrylic acid resin holder, strips of double-sided tape, epoxy, and a PEGylated coverslip as previously described^52^. The acrylic holder with dimensions of 76 mm × 26 mm × 5 mm (L × W × H), including the inlet and outlet holes, was fabricated with laser cutting. A PEGylated coverslip was placed on the double-sided tape attached to the acrylic holder, and the gaps were filled with epoxy. A yellow pipette tip was installed on an inlet port for a buffer reservoir, and tubing was connected to an outlet port by epoxy bonding that was cured at room temperature for 5 min. The dimensions of the flow chamber were 3 × 17 × 0.1 mm (L × W × H). A syringe pump, i.e., NE-1000 (New Era Pump Systems Inc., Wantagh, NY), was used to regulate the buffer delivery into the flow cell. After the preparation of the PEGylated surfaces, 25 μg mL^−1^ of NeutrAvidin in a T50 solution (10 mM Tris, 50 mM NaCl, pH 8.0) were loaded and incubated at room temperature for 5 min. One micromolar λ DNA overhang oligo (5′-pGGGCGGCGACCT-TEG-biotin-3′) was loaded into the flow cell and incubated at room temperature for 5 min. The mixture of λ DNA, T4 DNA ligase, and reaction buffer were loaded and kept at room temperature for 30 min. After washing the residual enzyme mixture with 1 × TE buffer (10 mM Tris, 1 mM EDTA, pH 8.5), the diluted DNA staining tTALE-FP (approximately 2 nM in 1 × TE buffer or NaCl salt buffer) flowed into the chamber and was incubated at room temperature for 5 min. The stained DNA molecules were visualized under a continuous flow of 1 × TE buffer or NaCl salt buffer, and the flow rate was maintained at 100 μL min^−1^.

### Flow cell with surface-immobilization

The flow chamber comprised an acrylic resin holder (slide glass size: 75 mm × 25 mm) and a positively charged cover glass. The acrylic resin holder was treated for 30 seconds in an air plasma generator (Cute Basic, FemtoScience, Suwon, Korea) to make the surface hydrophilic. A positively charged glass surface was placed on the double-sided tape (3 M) attached to the acrylic holder, and the gaps were filled with epoxy resin. A pipette was used for flushing DNA molecules, proteins, and buffer, as shown in Fig. 3E. DNA (1 pM) was loaded into a flow cell, and subsequently, a tTALE-FP solution was flushed to stain. After washing the residual proteins with 1 × TE solution, DNA was visualized with fluorescence microscopy. A lab-made python program has been used to analyze DNA images (Fig. 3F).

## Supplementary information

Movie S1

Movie S2

Movie S3

Movie S4

Movie S5
